# Static and dynamic stress analysis of different crown materials on a titanium base abutment in an implant-supported single crown: a 3D finite element analysis

**DOI:** 10.1186/s12903-024-04328-0

**Published:** 2024-05-10

**Authors:** Gonca Deste Gökay, Perihan Oyar, Gülsüm Gökçimen, Rukiye Durkan

**Affiliations:** 1 Department of Prosthodontics, Faculty of Dentistry, Bursa Uludag University, Bursa, Türkiye; 2 Dental Prosthetics Technology, School of Health Services, Hacettepe University, Ankara, Türkiye; 3 Department of Prosthodontics, Ankara 75Th Year Oral and Dental Health Hospital, Ankara, Türkiye; 4 Department of Prosthodontics, Faculty of Dentistry, Istanbul Okan University, Istanbul, Türkiye

**Keywords:** Material selection, Finite element analysis, Implant abutment, Biomechanics

## Abstract

**Background:**

This Finite Element Analysis was conducted to analyze the biomechanical behaviors of titanium base abutments and several crown materials with respect to fatigue lifetime and stress distribution in implants and prosthetic components.

**Methods:**

Five distinct designs of implant-supported single crowns were modeled, including a polyetheretherketone (PEEK), polymer-infiltrated ceramic network, monolithic lithium disilicate, and precrystallized and crystallized zirconia-reinforced lithium silicates supported by a titanium base abutment. For the static load, a 100 N oblique load was applied to the buccal incline of the palatal cusp of the maxillary right first premolar. The dynamic load was applied in the same way as in static loading with a frequency of 1 Hz. The principal stresses in the peripheral bone as well as the von Mises stresses and fatigue strength of the implants, abutments, prosthetic screws, and crowns were assessed.

**Results:**

All of the models had comparable von Mises stress values from the implants and abutments, as well as comparable maximum and minimum principal stress values from the cortical and trabecular bones. The PEEK crown showed the lowest stress (46.89 MPa) in the cervical region. The prosthetic screws and implants exhibited the highest von Mises stress among the models. The lithium disilicate crown model had approximately 9.5 times more cycles to fatique values for implants and 1.7 times more cycles to fatique values for abutments than for the lowest ones.

**Conclusions:**

With the promise of at least ten years of clinical success and favorable stress distributions in implants and prosthetic components, clinicians can suggest using an implant-supported lithium disilicate crown with a titanium base abutment.

## Introduction

Implant-supported prostheses are a common treatment choice in clinical dentistry because of their demonstrated functional, biological, and mechanical benefits as well as their long-term clinical success [1]. Because implants are in direct contact with bone and do not have periodontal ligaments, they exhibit biomechanical behaviors that are distinct from those of natural teeth. As a result, the occlusal stresses of the implant are immediately transmitted to nearby bone [2], potentially compromising the success of the implant [3]. Many factors, including the direction of loading [4] and the material and design parameters of the implant or restorative crown [5], influence the stress or energy transfer between the implant and peripheral bone. For improved biomechanical results, prosthetic dental components should be carefully chosen.

To combine the benefits of appropriate cosmetic qualities with the high mechanical performance of the titanium implant-abutment connection, a titanium base (Ti-base) was devised [6]. Implant-solution computer-aided design-computer aided manufacturing (CAD/CAM) blocks, which are ceramic blocks with a central hole used to facilitate connection with the Ti base and screw access to the implant, are necessary for the fabrication of the mesostructure. Without the mesostructure, the CAD/CAM blocks of the implant solution can also be machined as a crown. A one-piece design is a technique that simplifies the chairside process, uses a single CAD/CAM block, and enables screw-retained crown manufacturing [7].

Several materials have been suggested for use with monolithic ceramic restorations supported by implants. Lithium disilicate (LD) is a ceramic that comprises both lithium oxide and a vitreous phase, in contrast to polycrystalline ceramics such as zirconia, which are made up of sintered crystals without a vitreous matrix [8]. Recently, zirconia-reinforced lithium silicate (ZLS), which is based on lithium silicate and considerably reinforced with zirconia, was introduced. Additionally, it has shown good mechanical performance, either matching or surpassing LDs [9]. CAD-CAM ZLS blanks are available in a precrystallized (VITA Suprinity, VITA Zahnfabrik) or crystallized form (Celtra Duo, Dentsply Sirona). Resin-based materials such as polymer-infiltrated ceramic networks (PICNs), exemplified by Vita Enamic from Vita Zahnfabrik, are favored for their resilience against fracture and chipping, as well as their capacity to absorb chewing forces [10]. As indicated in the findings reported by Menini et al. [11], utilizing resin material for crown restoration can compensate for the high elastic modulus of the implant material, thereby diminishing the impact of the implant on the surrounding bone. A synthetic thermoplastic polymer is called polyetheretherketone (PEEK). It has been used as an alternative material for implant restorations due to its light weight, adequate fracture resistance, improved stress distribution, shock-absorbing qualities, and compatibility with different veneering materials [12].

Nonpolymeric or rigid frameworks with a high Young’s modulus have been reported to produce concentrated stress at the implant-bone interface [13, 14]. It has also been concluded that implant-supported crowns fabricated from resilient materials such as polymer-infiltrated ceramics and PEEK exhibit better force absorption than rigid materials such as zirconia and lithium disilicate ceramics [15]. Previous systematic reviews have shown that screw loosening is greater in single implant-supported crowns than in those with other prosthetic complications, followed by debonding and chipping [16, 17]. Monolithic ceramic implant-supported single crowns have been described as safe and effective options for short-term survival and low incidence of prosthetic complications, regardless of the ceramic material used [17]. Despite these results obtained from previous studies, the outcome of CAD-CAM crowns is still unknown, especially for implants in moderately high-force areas such as the premolar region. Given these considerations, researchers have directed their attention toward implants, particularly crown materials, to identify an optimal treatment approach and material.

Finite Element Analysis (FEA) is widely regarded as the most prevalent numerical method because it replicates the mechanical response under load based on a material's known properties. In two-dimensional (2D) or three-dimensional (3D) FEA software, parameters such as density, Poisson's ratio, and Young's modulus can be set based on the depth. 3D FEA, in particular, allows for the visualization of stress distribution throughout the entirety of implants and/or peripheral bone. The static load is the most commonly used load for FEA, but the dynamic impact is the actual force used in the mouth. Compared with static loading, dynamic loading increases stress levels and has greater osteogenic potential [18]. Thus, it is crucial to examine static and dynamic loading in stress analysis investigations [19]. Using 2D or 3D FEA, numerous studies have shown the behavior of implants rehabilitated with single crowns made with different materials. These studies used a static force to simulate the oral environment. Only a few studies have used 2D or 3D FEA to model dynamic forces under impact loading conditions [20].

The purpose of this study was to assess how the elastic modulus of materials affects the stress distribution in screw-retained implant-supported prostheses with Ti-base abutments under both static and dynamic loading. It has clinical relevance in terms of revealing which crown material will be more favorable for stress distribution to peri-implant tissues and present a longer lifespan of dental implants and prosthetic components. The initial null hypothesis states that the distribution of stress is not impacted by the various elastic characteristics of the crown materials. The second hypothesis states that the fatigue values of implants, abutments, crowns, and screws are not affected over time by the use of different crown materials.

## Materials and methods

To create a 3D FEA model of an implant-supported single crown with five different crown types in the maxillary right first premolar location, the current study was designed. SolidWorks software (version 2020, Dassault System, SolidWorks Corporation) was used to design and remodel virtual models of the bone level implant (Nobel Parallel Conical Connection TiUltra, Nobel Biocare AB) measuring 10.0 mm in length, 127 mm^2^ in grooved surface area and 3.75 mm in diameter; the Ti-base abutment (Universal base narrow platform, Nobel Biocare AB) measuring 3.0 mm in gingival height and 3.75 mm in diameter; the retaining screw (Universal base screw, Nobel Biocare AB) measuring 3.9 mm in length; and five distinct restorative crowns. A 3D model was created to simulate a one-piece prosthetic solution composed of a crown over a Ti base. The interface of the Ti-base abutment and implant crown was completely bonded without any gap, and the cement layer was excluded [21]. After that, this set was screwed onto a conical connection implant.

A segment of maxillary bone in the premolar area was generated and subsequently processed using Rhinoceros 4.0 (3670 Woodland Park Ave N, Seattle, WA 98103 USA). Trabecular bone was acquired by referencing the inner surface of the maxillary cortical bone, which was adjusted to a thickness of 2 mm.

**Fig. 1:**
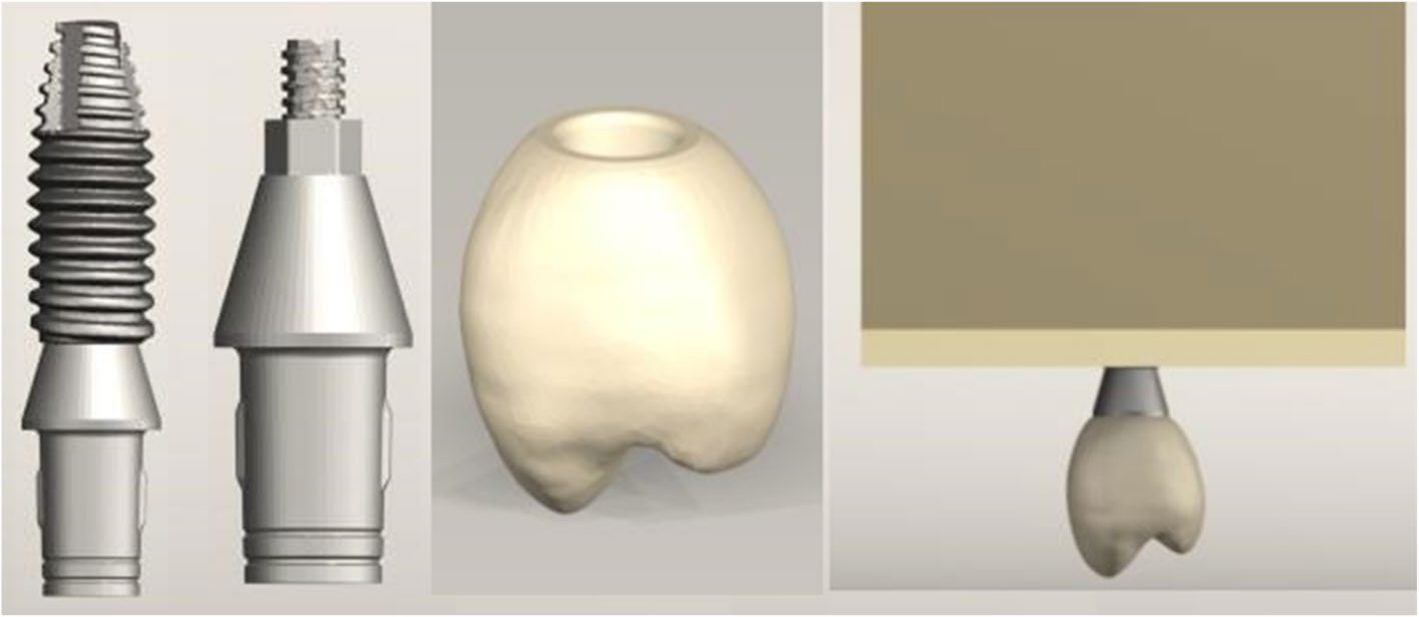
3D modeling of the implant, titanium base abutment and crown and placement in the alveolar bone segment

A right maxillary first premolar with dimensions of 1.5 mm in width for the central fossa, 9 mm in width for the buccopalatal, 8 mm in width for the mesiodistal, and 8.5 mm in height for the crown was positioned on the Ti-base abutment per the restorative crown. The five crown types—a crystallized ZLS (Celtra Duo, Densply Sirona), a precrystallized ZLS (Vita Suprinity, Vita Zahnfabrik), a PICN (Vita Enamic, Vita Zahnfabrik), a lithium disilicate (IPS e.max CAD, Ivoclar Vivadent), and PEEK (JUVORA, Invibo)—were combined with a Ti-base abutment to create the hybrid abutment-crown. The five models were created using various crown types (Fig. 1). The coefficient of friction between the implant, abutment, and screw in the implant system was determined to be 0.3 [22]. The dental implant was placed in the simulated bone with complete osseointegration. All other contacts were considered rigidly bonded.

**Fig. 2:**
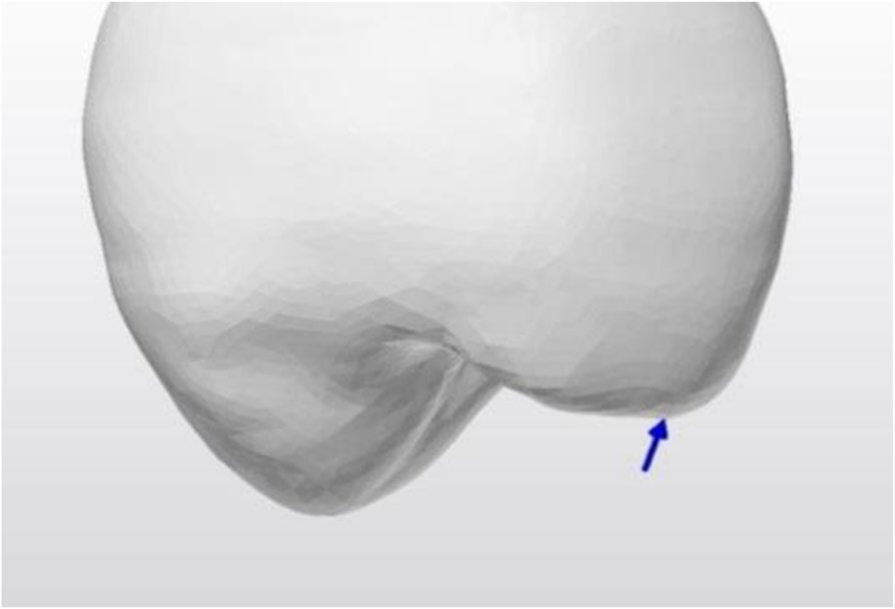
The direction and position of the applied load

The values of Poisson's ratio, Young's modulus (elastic modulus) and yield strength were taken from previous studies (Table 1) [21, 23–27]. Boundary conditions limited the movement of the surrounding bone along the x-, y-, and z-axes. For each of the 3D models, a discretization process involving ten nodes of quadratic tetrahedral components was carried out using meshing software (VRMesh Studio; VirtualGrid, Inc.). For each model, 167 219 nodes and 901 978 tetrahedral elements were employed. To ensure that the amount of mesh would not affect the study's findings, these models were created using a 10% mesh convergence test [28]. The FEA program (Algor Fempro; ALGOR) was utilized to evaluate the stress distribution on the mesh models. All the models were assumed to be linearly elastic, homogenous, and isotropic. For static loading [29], a 100 N oblique load of 30 degrees was applied to the buccal incline of the palatal cusp of the maxillary right first premolar (Fig. 2).

**Table 1:** Mechanical properties of the materials used in this study

**Materials**	**Elastic modulus (GPa)**	**Poisson's ratio**	**Ultimate tensile Strength (MPa)**
Cortical bone	13.7	0.30	-
Trabecular bone	1.37	0.30	-
Titanium (implant and prosthetic screw)	110.0	0.35	825
Titanium base abutment	110.0	0.35	825
Crystallized zirconia-reinforced lithium silicate (ZLS)	107.9	0.222	159
Precrystallized zirconia-reinforced lithium silicate (ZLS)	104.9	0.208	180
Lithium disilicate (LD)	102.7	0.215	173
Polymer-infiltrated ceramic network (PICN)	37.8	0.24	227
Polyetheretherketone (PEEK)	3.5	0.36	80

The dynamic loading was carried out in the same manner as the static loading with a frequency of 1 Hz [30]. The stress values found during static loading were imported into the fatigue Wizard program (Autodesk, Algor Fempro, Algor) prior to the computation of the dynamic loading results (number of cycles until fatigue failure). The fatigue lifetime was computed using 350,000 chewing cycles, which is the average number of chewing cycles per individual per year, and 10^7^ cycles corresponding to a 30-year lifespan for all fatigue failure analyses [31, 32].

Von Mises stresses within the implant-crown complex as well as the maximum and minimum principal stresses in the peri-implant bone structure were determined. In the FEA, color coding of the stress distribution made it possible to compare the biomechanical differences between models. Stress concentration theories were used to acquire fatigue failure data or determine the effects of dynamic force application [31, 32].

## Results

Table 2 displays the results of the von Mises stress analyses. All of the models had comparable von Mises stress values from the implants and abutments, as well as maximum and minimum principal stress values from the cortical and trabecular bones.

**Table 2:** von Mises stress values (MPa) observed in five different experimental groups

	**Implant**	**Abutment**	**Screw**	**Crown**
**Precrystallized ZLS model**	704.28	404.81	1353.99	171.54
**Crystallized ZLS model**	704.28	404.81	1353.98	173.10
**PICN model**	704.62	405.14	1351.45	141.52
**LD model**	704.30	404.82	1353.91	171.79
**PEEK model**	705.24	405.62	1346.97	46.89

**Fig. 3:**
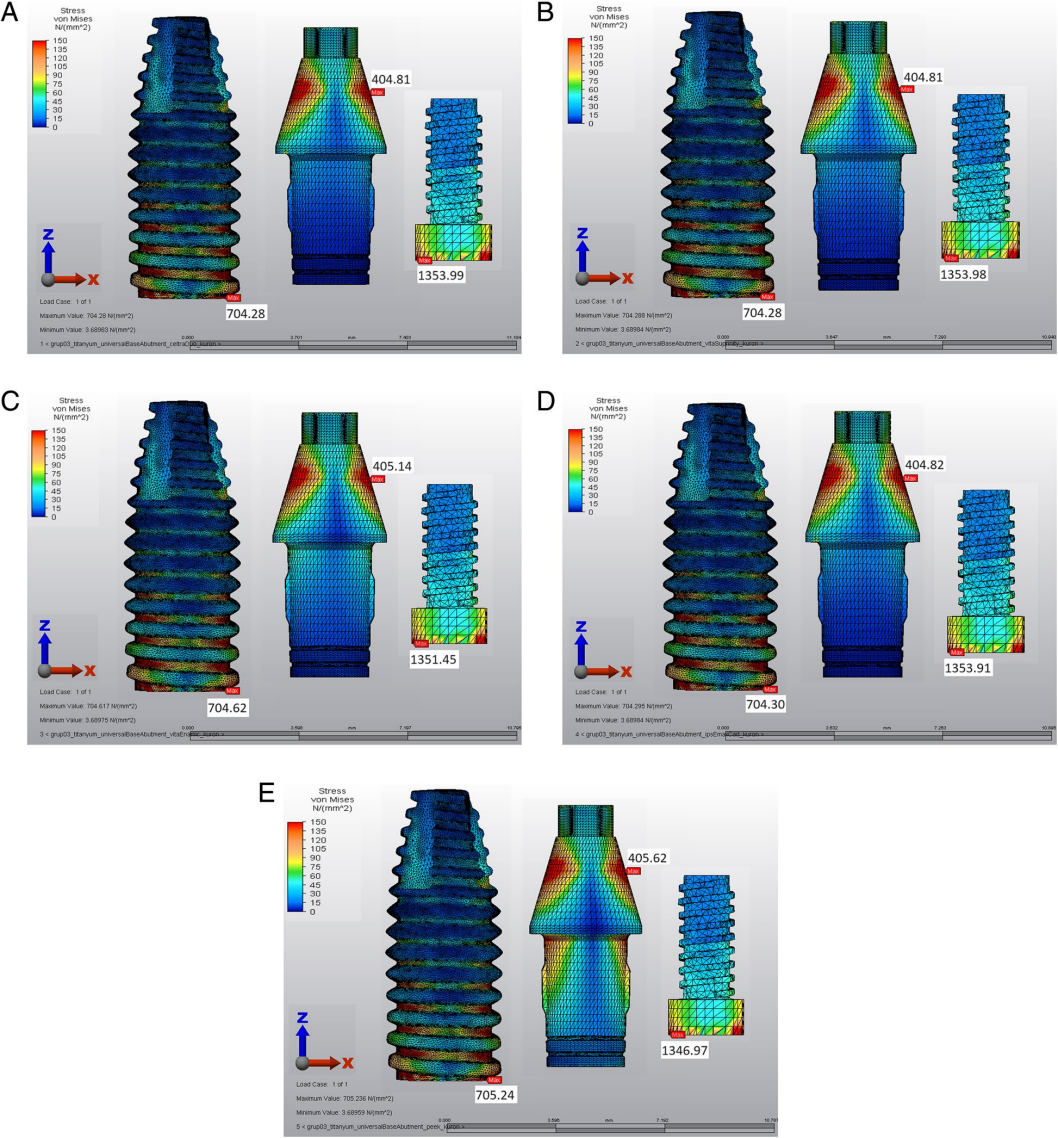
von Mises stresses of the implant, abutment and screw. **A** Precrystallized ZLS crown model, **B** Crystallized ZLS crown model, **C** PICN crown model, **D** LD crown model, **E** PEEK crown model

Across all the models, the von Mises stress was highest, ranging from 1353 to 705 MPa, for both the prosthetic screws and the implants. The first thread in the palatal neck region of the implants contained the majority of the von Mises stresses. The mesiobuccal region of the screw head was the focal point of the von Mises stresses in the prosthetic screw. The gingivocervical region was found to be the location of the von Mises stress on the Ti-base abutments, with the palatal surface exhibiting the highest values (Fig. 3).

In general, the cortical bone exhibited greater maximum (28.88 MPa) and minimum principal stress values (-49.3 MPa) than did the cancellous bone (2.72 and -3.21 MPa). Similarly, in all the models, the patterns and locations of the stress concentrations were comparable. The mesiobuccal region of the cortical bone and the distopalatal region of the trabecular bone were the locations of the maxillary principal stresses. The palatal region of the cortical and trabecular bones was the focal point of the minimum principal stresses.

**Fig. 4:**
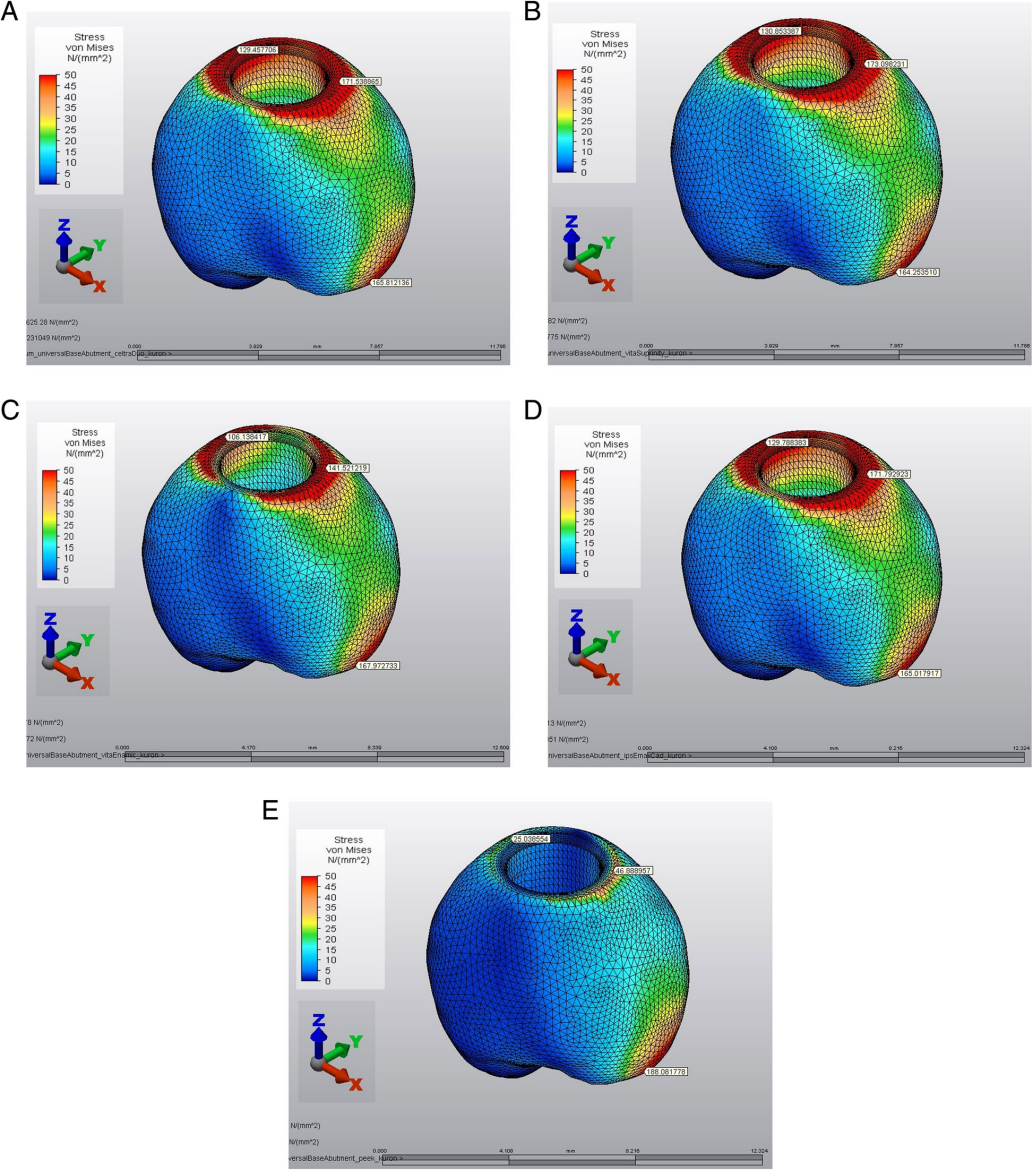
von Mises stresses of implant-supported crowns. **A** Precrystallized ZLS crown model, **B** Crystallized ZLS crown model, **C** PICN crown model, **D** LD crown model, **E** PEEK crown model

In all the restorative crowns, the von Mises stresses were localized in the palatal region of the marginal finish area. The PEEK crown showed the lowest stress values in the cervical region. The stress levels in the PICN were lower than those in the ZLS and LD crowns (Fig. 4).

**Fig. 5:**
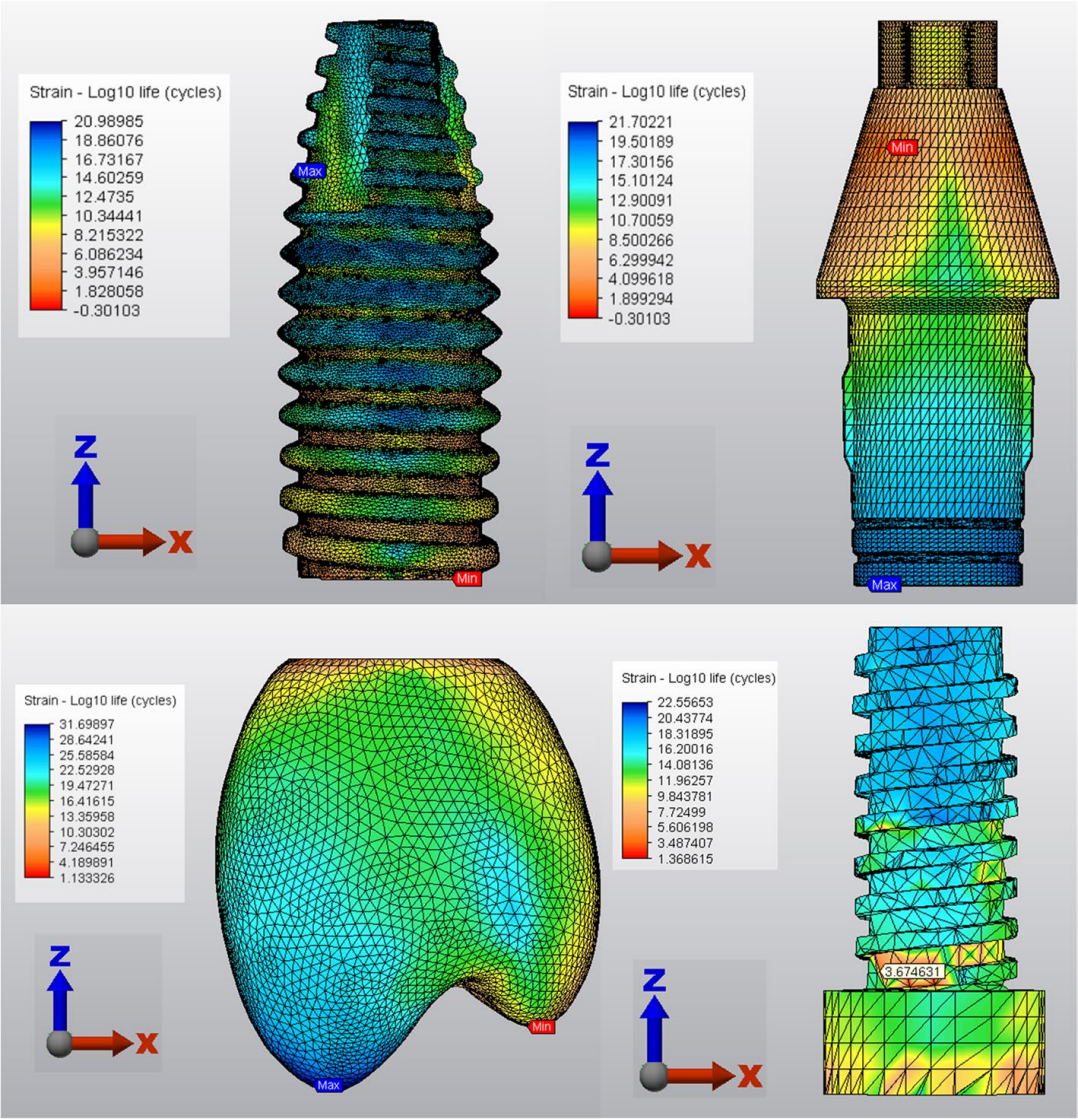
Cycle of fatigue failure in the lithium disilicate crown model

The performances of the prosthetic components and implants in the various crown models are compared in Table 3. The lowest number of cycles to fatigue failure and the shortest fatigue lifetime were found in the PEEK crown model for implants and the PICN crown model for abutments. The number of cycles to fatigue failure and fatigue lifetime were similar for the LD, PEEK and PICN crowns. Both ZLS crown models demonstrated the lowest number of cycles to fatigue failure and the shortest fatigue lifetime for prosthetic screws. Compared with those of the ZLS crown models, the cycles to fatigue values were 3.5 times greater for the other crown models for prosthetic screws. The LD crown model exhibited at least ten years of clinical success for implants, abutments, screws and crowns (Fig. 5).

**Table 3:** Cycles of fatigue failure/fatigue lifetime (in years) of the five experimental models

	**Crown**	**Implant**	**Abutment**	**Screw**
**Precrystallized ZLS model**	5.00/14.29	4.25/12.15	2.89/8.26	1.00/2.87
**Crystallized ZLS model**	5.00/14.29	1.44/4.13	8.99/25.69	1.00/2.87
**PICN model**	5.00/14.29	1.42/4.06	2.37/6.76	3.60/10.30
**LD model**	5.00/14.29	9.77/27.91	5.04/14.39	3.60/10.29
**PEEK model**	7.07/20.19	1.01/2.90	2.97/8.47	3.61/10.31

## Discussion

The crown that is placed over the implant needs to be strong enough to resist chewing forces without rupturing the implant or the tissue around it. The tendency of a dental crown to deform under stress is known as its elastic modulus, and it can be crucial to the outcome of implant therapy. The impact of five distinct implant-supported monolithic restorative materials on fatigue lifetime and biomechanical behavior was assessed in this study. The stress distribution throughout the implant-bone structure was not affected by the type of crown material. The null hypothesis, which states that the distribution of stress is not impacted by the various elastic characteristics of the crown materials, was accepted in light of the results.

According to Sevimay et al. [21] and Vieira et al. [33], the use of more elastic or rigid materials has an impact on the localization and distribution of stress in the abutment and crown, but it has no effect on the amount or distribution of stress in the bone tissue. In a study, the stress distribution in LD ceramic and resin nanoceramic CAD/CAM crowns was compared under vertical loading, and it was reported that resin nanoceramic crowns showed lower stress values [34]. In the current study, even with different crown materials, similar stress distributions were observed throughout the model. This different result can be attributed to the use of oblique loading in the current study. However, differences were observed in the stress distribution in the crown marginal end area. Wang et al. [35] also reported that the load distribution in bone tissue was not significantly affected by different prosthetic materials (gold alloy, resin, or porcelain) in a single implant-supported crown. While the displacement may differ for various crown materials under a load, the overall energy transferred to both the abutment-implant interface and subsequently to the implant-bone interface remains comparable [36]. Tribst et al. [37] discovered that the distribution of stresses in bone did not differ among zirconia, LD, and hybrid ceramic crowns. Another study assessed the biomechanical behavior of customized PEEK abutments and resin matrix ceramics. It was discovered that restorative materials did not affect the distribution of stress in the implant or surrounding bone and that crown stress values were lower in resin-infiltrated glass–ceramics [29]. Similarly, PEEK and glass–ceramics had the lowest stress values in crowns in the present study. These materials may concentrate less stress in their structure due to their low elastic modulus. In parallel with this result, the PEEK crown had a longer fatigue lifetime. A PEEK crown with an elastic modulus almost 30 times lower than that of the ZLS crown and an almost 2 times lower tensile strength demonstrated the longest fatigue lifetime for crown structures but also generated the shortest fatigue lifetime for dental implants. Compared to those of other crown materials, the PEEK crown had approximately 1.5 times more cycles to fatigue failure. This is because the higher the crystallinity of the crown material is, the more the stress is concentrated within the material. A stiffer foundation substrate resulted in higher fatigue performance of the restorative set [38].

High 5- to 10-year success rates for single-implant crowns, ranging from 95 to 97%, have been demonstrated in numerous clinical investigations [39, 40]. The current study yielded comparable results, with each restorative crown material demonstrating a fatigue lifetime exceeding ten years.

Failures in the implant-abutment connection may be associated with the use of crowns with a low elastic modulus. However, in a dynamic FEA study, it was found that the risk of bone resorption around the implant can be reduced by using more elastic materials that can better distribute the impact energy and reduce the stress transferred to the implant [20]. Mourya et al. [41] showed that the use of a crown material with a lower elastic modulus, such as PEEK crowns, reduced the stress concentration in bone compared to porcelain fused to a metal crown. It was concluded that the PEEK crown and straight implant abutment prevent possible implant failure. In contrast, in the present study, the PEEK and PICN crown models, in which lower elastic modulus crown material was used, had the shortest fatigue life for the implant and abutment. In the abovementioned study, the bruxism scenario was simulated using a vertical 1000 N and oblique 500 N load, a Co-Cr metal framework with a higher elastic modulus was used for comparison, and only static stress analysis may have led to these different results. Compared to the models with the shortest fatigue lifetime, the LD crown model had a fatigue lifetime approximately 1.7 times longer for abutments and 9.5 times longer for implants. The second hypothesis of the current study was disproven since the results of the investigation indicate that different types of crown material had an effect on fatigue values after dynamic loading. From this perspective, the impact of restorative material on restorative set performance is not novel and can be explained by the microstructure and mechanical characteristics of dental ceramics, which are closely linked to the behavior of the materials under stress [42].

For the cortical bone, the critical compressive and tensile stress values are 167–205 MPa and 121–135 MPa, respectively [43]. In the present study, the maximum and minimum principal stress values were approximately 29 and -49 MPa, respectively. The total stress values obtained were less than the ultimate tensile and compressive strength of the cortical bone. The findings of the present study support the findings of previous studies in that stress was primarily localized in the cortical bone surrounding the implant neck area rather than in the apical region [13, 44]. In the present study, as in many FEA studies, a high stress concentration was observed in the first thread of the implant [45–47].

In clinical settings, increased stresses may result in screw loosening and fracture, bone loss, loss of osseointegration, and prosthesis or implant fracture [48, 49]. Because loosening of the prosthetic screw often occurs, this should be considered [50]. According to previous research, screw loosening occurs in 3.91–17% of cases [51]. In the present study, the highest von Mises stresses were concentrated in the prosthetic screw, and the screw was the most susceptible to fatigue. Compared with those of the ZLS crown models, the cycles to fatigue values were 3.5 times greater for the other crown models for prosthetic screws.

According to the fatigue failure analysis, there was a high risk of complications associated with screw deformation in the ZLS crown models. However, the Ti-base was designed with easy retrievability, and the risk of Ti-base deformation at the implant-abutment connection in the precrystallized ZLS crown model was lower than that in the crystallized ZLS crown model. The present study determined the fatigue limit of the implant-abutment-crown complex for each group, that is, the fatigue lifetime of a model. This information is more useful than the conclusions that can be drawn from static FEA.

Determining the biomechanical behavior and fatigue lifetime of different implant crowns on a Ti-based abutment under static and dynamic loads can guide clinical indications and material selection. According to the results of this 3D FEA study, maxillary premolar implant-supported lithium disilicate crowns with a Ti-base abutment can be recommended for clinicians, as these crowns are promising for at least 10 years of clinical success in implants and prosthetic components. A cohort study also reported that the 10-year survival and chipping-free rate of LD implant-supported crowns were 93.8% [52]. The combination of the ZLS crown and Ti-base abutment should be used provided that clinicians check the prosthetic screw regularly. However, long-term follow-up studies are needed to confirm the present results and to report the success and survival rates of premolar implant crowns.

This study has limitations, notably in the context of FEA. It is essential to recognize the limitations inherent in FEA, and validation through clinical investigation is necessary. The exact geometry of the implant and its components—especially the crown materials—as well as the real material qualities and clinical settings are not the same in FEA. Further research is needed to determine the prosthetic screw micromotion or displacement to explain screw loosening complications. The results of the present study could improve the understanding of the biomechanical aspects of selecting the optimal material for an implant-supported crown and predicting issues.

## Conclusion

The combination of a Ti-base abutment and a stiff crown lengthened the functional lifetime of the screw, abutment, and implant according to the results of this static and dynamic FEA analysis. The stress distribution throughout the implant-bone structure was unaffected by the type of crown material.

## Data Availability

The datasets used and/or analyzed during the current study are available from the corresponding author upon reasonable request.

## Abbreviations

2D: Two-dimensional
3D: Three-dimensional
PEEK: Polyetheretherketone
Ti-base: Titanium base
CAD/CAM: Computer-aided design/Computer-aided manufacturing
LD: Lithium disilicate
ZLS: Zirconia-reinforced lithium silicate
PICN: Polymer-infiltrated ceramic network
FEA: Finite element analysis
